# mCLCA3 Modulates IL-17 and CXCL-1 Induction and Leukocyte Recruitment in Murine *Staphylococcus aureus* Pneumonia

**DOI:** 10.1371/journal.pone.0102606

**Published:** 2014-07-17

**Authors:** Kristina Dietert, Katrin Reppe, Lars Mundhenk, Martin Witzenrath, Achim D. Gruber

**Affiliations:** 1 Department of Veterinary Pathology, Freie Universität, Berlin, Germany; 2 Department of Infectious Diseases and Pulmonary Medicine, Charité – Universitätsmedizin, Berlin, Germany; Louisiana State University, United States of America

## Abstract

The human hCLCA1 and its murine ortholog mCLCA3 (calcium-activated chloride channel regulators) are exclusively expressed in mucus cells and linked to inflammatory airway diseases with increased mucus production, such as asthma, cystic fibrosis and chronic obstructive pulmonary disease. Both proteins have a known impact on the mucus cell metaplasia trait in these diseases. However, growing evidence points towards an additional role in innate immune responses. In the current study, we analyzed *Staphylococcus aureus* pneumonia, an established model to study pulmonary innate immunity, in mCLCA3-deficient and wild-type mice, focusing on the cellular and cytokine-driven innate inflammatory response. We compared clinical signs, bacterial clearance, leukocyte immigration and cytokine responses in the bronchoalveolar compartment, as well as pulmonary vascular permeability, histopathology, mucus cell number and mRNA expression levels of selected genes (mClca1 to 7, Muc5ac, Muc5b, Muc2, Cxcl-1, Cxcl-2, Il-17). Deficiency of mCLCA3 resulted in decreased neutrophilic infiltration into the bronchoalveolar space during bacterial infection. Only the cytokines IL-17 and the murine CXCL-8 homolog CXCL-1 were decreased on mRNA and protein levels during bacterial infection in mCLCA3-deficient mice compared to wild-type controls. However, no differences in clinical outcome, histopathology or mucus cell metaplasia were observed. We did not find evidence for regulation of any other CLCA homolog that would putatively compensate for the lack of mCLCA3. In conclusion, mCLCA3 appears to modulate leukocyte response via IL-17 and murine CXCL-8 homologs in acute *Staphylococcus aureus* pneumonia which is well in line with the proposed function of hCLCA1 as a signaling molecule acting on alveolar macrophages.

## Introduction

The human hCLCA1 and its murine ortholog mCLCA3 are members of the CLCA (calcium-activated chloride channel regulator) family with a well established role in inflammatory airway diseases with increased mucus production such as asthma, cystic fibrosis (CF) and chronic obstructive pulmonary disease (COPD) [1]–[4]. The link between CLCA proteins and inflammatory airway diseases has been recognized based on overexpression of Clca gene products in affected airways which is regulated by Th2 cytokine signals (IL-4, IL-9, and IL-13) [4], [5]. The secreted proteins hCLCA1 and mCLCA3, which are selectively expressed in mucus cells of airways and other tissues [6]–[8] have been directly linked to the trait of mucus cell metaplasia in inflammatory airway diseases [9]. Specifically, it was previously demonstrated that hCLCA1 acts as an extracellular signaling protein, inducing mucus gene transcription via a downstream mitogen-activated protein kinase (MAPK)-13 signaling pathway and hereby regulating mucus cell metaplasia [10]. Hence, hCLCA1 and its ortholog mCLCA3 have been proposed as biomarkers of inflammatory airway diseases [11] and as targets for therapeutic intervention in mucus overproduction [11], [12].

However, in addition to the modulation of mucus production and the strong link to mucus cell metaplasia, CLCA-proteins have been implicated in the regulation of tissue inflammation in the innate immune response [13], [14]. Indeed, recent studies have demonstrated that hCLCA1 may act as an innate immune signaling molecule which activates airway macrophages and thereby enhances pro-inflammatory cytokine release (IL-8, IL-6, IL-1β, TNF-α) [15]. Moreover, asthmatic mice treated with anti-mCLCA3-antibodies showed remarkable reduction of airway inflammation [16].

So far, only models of chronic and allergic airway inflammation [9], [13], [17] and acute inflammation due to LPS [13] have been characterized in mCLCA3-deficient mice. However, acute bacterial infection appears more suitable to test for a role of mCLCA3 in modulating innate immune responses.

Consequently, this study adopted infection of mCLCA3-deficient mice with *Staphylococcus aureus* (*S. aureus*) which is one of the most prevalent pathogens of community- and hospital-acquired infections in humans accounting for a significant health and economic burden [18]–[20]. Besides septicemia, skin and soft tissue infections, *S. aureus* causes lower respiratory tract infections in humans, especially in infants and young children with CF [21]–[23].

Here, we hypothesized that mCLCA3 has an impact on the innate immune response in acute *S. aureus* infection of the lung. mCLCA3-deficient mice (mClca3^−/−^) and wild-type (WT) littermates were infected with *S. aureus* and the course of pneumonia was analyzed in comparison with uninfected mice regarding clinical signs, bacterial clearance, leukocyte immigration and cytokine response in bronchoalveolar lavage fluid (BALF), pulmonary vascular permeability, histopathology including morphometry, mucus cell quantification and respiratory tract mRNA expression levels of selected genes of interest, including mClca1 to 7, Muc5ac, Muc5b, Muc2, Cxcl-1, Cxcl-2 and Il-17. We show that mCLCA3 modulates the cellular leukocyte recruitment via IL-17 and CXCL-1 in bacterial pneumonia and thus appears to have an impact on the early innate immune response following *S. aureus* lung infection.

## Materials and Methods

### Ethics Statement

All animal procedures were approved by the ethics committee of the Charité - Universität Berlin and local governmental authorities (Landesamt für Gesundheit und Soziales Berlin, approval ID: G 0358/11). The animal studies were conducted in strict accordance with the FELASA guidelines and recommendations for the care and use of laboratory animals.

### Mice

Female mCLCA3-deficient mice (mClca3^−/−^) and C57BL/6J WT littermates (aged 8–9 weeks and weighing 18–20 g) were randomly assigned to groups of four and housed in individually ventilated cages under SPF conditions with a room temperature of 22±2°C and a relative humidity of 45%–65%. A 12 hour light/12 hour dark cycle was maintained and the animals had unlimited access to standard pelleted food and tap water. mClca3^−/−^ mice were generated on a C57BL/6J background by replacing parts of exons 7–11 with a neomycin cassette [9]. For all experimental procedures, excluding the infection procedure, mice were anesthetized by intraperitoneal injection of premixed ketamine (3.2 mg) and xylazine (1.5 mg) and sacrificed by exsanguination via the caudal *Vena cava*.

### Bacterial Growth and Infection

*Staphylococcus aureus* Newman (*S. aureus*) from –80°C glycerol stocks was plated on Columbia agar with 5% sheep blood and incubated overnight at 37°C with 5% CO_2_. Following incubation, single colonies were picked and incubated in 25 ml Caso-bouillon (Carl Roth, Karlsruhe, Germany) for 2 to 3 hours at 220 rpm until midlog phase (A_600_ = 0.5). After centrifugation at 800×g, the pellet was resuspended in sterile PBS to an adjusted infectious dose (5×10^7^ cfu *S. aureus*). For infection, mice were anesthetized by intraperitoneal injection of premixed ketamine (1.6 mg) and xylazine (0.5 mg) and transnasally inoculated [24] with 5×10^7^ cfu *Staphylococcus aureus* Newman in 20 µl sterile PBS. Controls received 20 µl of sterile PBS. Body weight and rectal temperature (BAT-12 Microprobe, Physitemp, Clifton, NJ, USA) were recorded every 12 hours.

### Bacterial Burden in Lungs, Liver and Blood

Mice were intraperitoneally anesthetized, heparinized, and blood was taken from the caudal *Vena cava*. Sterilely dissected lungs and livers were homogenized, serial dilutions of homogenates and blood were plated on blood agar and incubated (37°C, 5% CO_2_) for 24 h for subsequent counting of colony forming units.

### Quantification and Differentiation of Leukocytes in BALF and Blood

At indicated time points, mice were intraperitoneally anesthetized, tracheotomized, ventilated and intracardially heparinized as described [24]. Blood was taken from the caudal *Vena cava* and lungs were perfused with 0.9% NaCl via the pulmonary artery. Bronchoalveolar lavage was performed twice with 800 µl ice-cold PBS. After spinning, supernatant was snap frozen for cytokine analyses. Total leukocytes were counted manually on Neubauer Chamber and differentiated by fluorescent-activated cell sorter (FACS) analysis (FACS Calibur, BD Biosciences, Heidelberg Germany) using forward versus side scatter characteristics and the specific antibodies CD45 PerCP (clone 30-F11, BD Biosciences), GR-1 PE (clone RB6-8C5, BD Biosciences) and F4-80 APC (clone BM8, Invitrogen, Karlsruhe, Germany) as described [25]. Total blood leukocytes were counted and differentiated by FACS analysis using BD TruCOUNT Tubes, forward versus side scatter characteristics and specific antibody staining with CD45 PerCP and GR-1 PE [25]. Cytospins from BALF were obtained by centrifugation of 100 µl BALF cell suspension at 20×g for 10 minutes (Cytospin 3, Shandon Ltd, Runcorn, UK) and subsequently stained with May-Grünwald Giemsa.

### Pulmonary Vascular Permeability and Protein Quantification

Mouse albumin (MA) was quantified in BALF and plasma by ELISA (Biomol, Hamburg, Germany) according to the manufacturer’s instructions. Pulmonary vascular permeability was assessed by calculating the MA BALF/plasma ratio. Total protein of BALF was measured via bicinchoninacid-assay (BCA) according to the manufacturer’s instructions using Pierce BCA Protein Assay (Thermo Scientific, Rockford, USA).

### Quantification of Cytokines in BALF

Cytokines (IL-1β, IL-6, IL10, IL12p40, IL-13, MCP-1, GM-CSF, RANTES, CXCL-1 and TNF-α) were measured in the supernatant of BALF using a cytokine protein multiplex assay (Bioplex, Bio-Rad, Hercules, CA) and IL-17 was quantified by ELISA (ab100702, Abcam, Cambridge, UK) according to the manufacturer’s instructions.

### RNA Isolation and Quantitative RT-PCR

Total RNA was isolated from lungs and trachea using Nucleo Spin RNA/Protein isolation Kit (Macherey Nagel, Düren, Germany). Total isolated RNA was quality checked and quantified using the NanoDrop ND-100 Spectrophotometer (Peqlab, Wilmington, USA). Transcript expression levels of murine Clca1 to 7, Muc5ac, Muc5b, Muc2, Il-17, Cxcl-1 and Cxcl-2, normalized to the reference genes elongationfactor 1α (Ef-1α), β-2 microglobulin (B2m) and glyceraldehyde-3-phosphate dehydrogenase (Gapdh), were determined. For exact transversion, 100 ng of RNA from lungs and trachea were reverse transcribed twice, each time in 20 µl reaction volume using Iscript (BioRad) according to manufacturer’s instructions. Primers and probes for mClca1, mClca2, mClca3, mClca5 mClca6, mClca7, Ef1-α [26], Muc5ac, Muc5b [17], Muc2 [27], Il-17, Gapdh [28] and B2m [29] were used as described. Primers and probes for mClca4, Cxcl-1 and Cxcl-2 were designed using Primer3 software (WWW primertool, Whitehead Institute of Biomedical Research). All primer pairs encompassed an intron to avoid amplification of genomic DNA. In silico analysis of primer sequences and probes using NCBI nucleotide BLAST searches revealed 100% identity only with the expected DNA sequence. Agarose gel electrophoresis and sequencing of all PCR-products confirmed an amplification of a single product of expected size and sequence. For mClca4, no cross-reactions were detected when linearized, fully cloned cDNA samples of mClca1 and mClca2 were used as templates. Gapdh, Ef1-α and B2m were used as internal reference genes. Primer and probe sequences as well as amplicon sizes and annealing temperatures are listed in Table S1. An optimized reaction-mix in a total volume of 15 µl containing Maxima Probe qPCR Master Mix (Thermo Scientific, Oxford, USA), forward and reverse primers (each 0.3 µM), probe (0.2 µM), nuclease free water to adjusted volume as well as 1 µl of template cDNA was applied and cycling conditions according to manufacturer’s instructions were used. RT-qPCR protocols were established using 10 fold serial dilutions ranging from 10^2^ to 10^6^ copies of purified (Nucleo Spin Gel and PCR Clean-up, Macherey-Nagel) PCR-derived fragments of each mRNA. RT-qPCR and data analyses were conducted using the CFX96 Touch Real-Time PCR Detection System and CFX Manager software 1.6 (BioRad). Relative quantification and comparison of groups were performed by the ΔΔCt method using uninfected wild-type animals as controls.

### Western Blot Analysis

BALF proteins were separated by denaturating SDS (10%) PAGE followed by immunoblotting with mCLCA3 specific antibodies as described [6].

### Histology and Immunohistochemistry

In a separate set of experiments, mice were intraperitoneally anesthetized, heparinized and blood was taken from the caudal *Vena cava*. After careful removal, lungs were immersion-fixed in 4% formalin (pH 7.0) and processed as described [6]. Briefly, sections were stained with hematoxylin and eosin and periodic acid-Schiff (PAS) reaction was performed. Three evenly distributed sections from each lung were scored for various parameters [30]–[32] (Table 1), and PAS-positive cells per millimeter of basement membrane were counted. *S. aureus* was detected by immunohistochemistry as described [33].

**Table 1:** Lung Scoring Parameters.

Parameter	Scale/score
**Lung inflammation:**	
Bronchitis	0–4
Peribronchial inflammation	0–4
Interstitial inflammation	0–4
Intraalveolar inflammation	0–4
Alveolar necrosis	0–4
Alveolar edema	0–4
Perivascular edema	0–4
Perivascular inflammation	0–4
Infiltration by neutrophils	0–4
Infiltration by macrophages	0–4
**Lung area affected**	0–100%

### Lung Morphometry for Volume Estimation by the Cavalieri Principle

Total volume of left lungs from infected mice and corresponding lesions were assessed by Cavalieri principle [34], [35]. Left lungs of *S. aureus* infected mice (24 hours post infection, n = 4) were cut into 140 sections of equal thickness (10 µm). Each seventh section was stained with hematoxylin and eosin for subsequent determination of total cut face areas (A_CF_) as well as total areas of lung lesions (A_L_) using digital image analysis software (AnalySIS docu 5.0., SIS). An estimated volume of the left lungs and of left lung lesions was calculated by multiplying the slab thickness (*T = 10* *µm×7*) with the sum of the cut face areas (A_CF_) or lesion areas (A_L_) of each particular section (*V = T×*∑ *A_CF_*; *V = T×∑A_L_*) [34]–[36]. Finally, the percentages of lesions were calculated.

### Data Analysis

Data are expressed as mean ± SEM. Statistical analyses were performed using the Mann-Whitney test. P<0.05 was considered significant.

## Results

### mCLCA3 deficiency had no impact on clinical outcome of *Staphylococcus aureus* pneumonia

**Figure 1:**
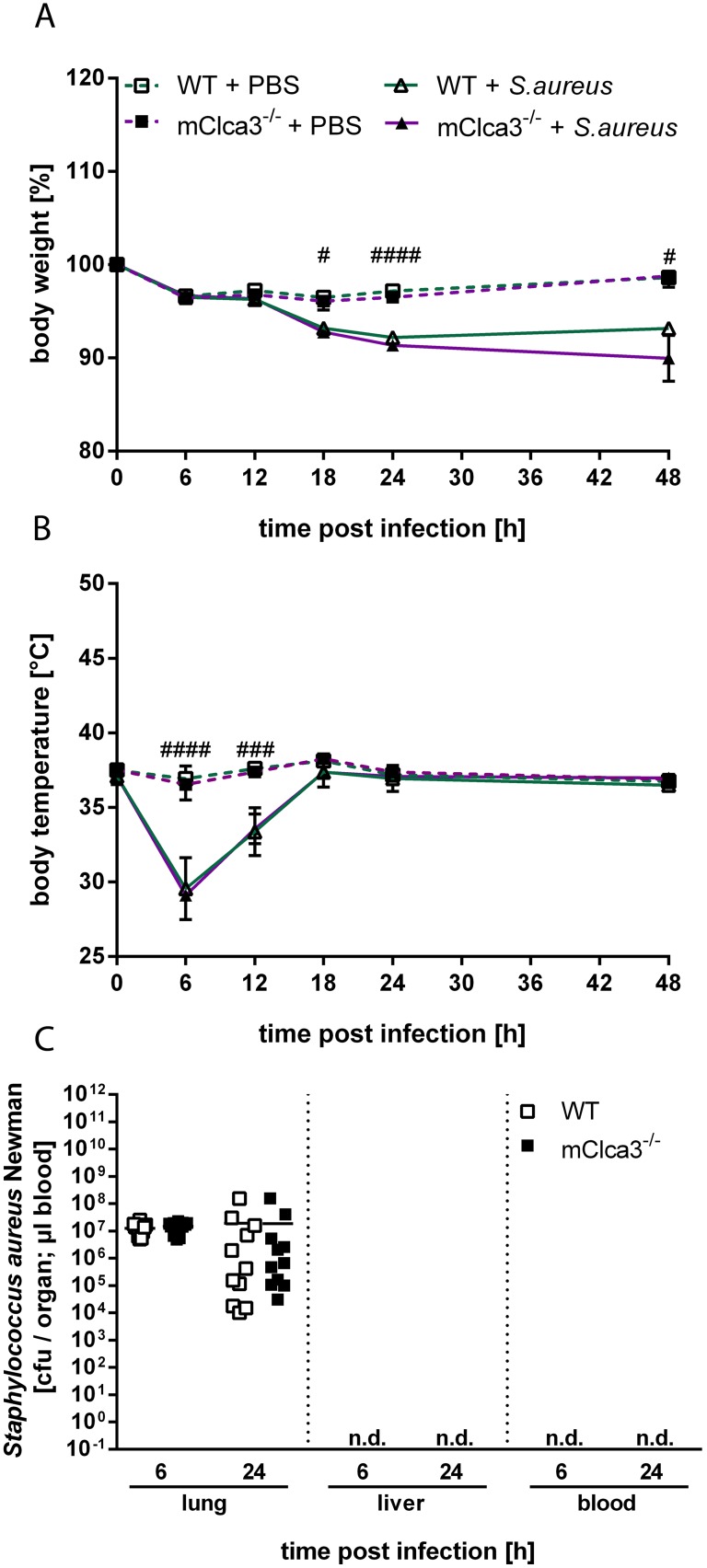
mCLCA3-deficiency had no impact on clinical outcome of pneumonia or bacterial loads in lungs. mClca3^−/−^ and WT mice were transnasally infected with 5×10^7^ *Staphylococcus aureus* Newman or received PBS (controls) and body weights (A) and temperatures (B) were measured every 6 hours for 24 hours and once after 48 hours. Values are given as mean ± SEM (n = 8 each group). ^#^p<0.05, ^##^p<0.01, ^###^p<0.001, ^####^p<0.0001 versus the PBS control group. (C) Lung, liver and blood bacterial loads were determined 6 hours and 24 hours after infection. Values are given as individual data and mean (n = 11 each group). n.d.  =  not detected.

After infection of mCLCA3-deficient and wild-type mice with *S. aureus* Newman, both genotypes equally developed acute pneumonia clinically hallmarked by significant loss of body weight (Figure 1A) and temperature (Figure 1B) compared to PBS controls. Infected animals showed similarly constant bacterial loads after 6 and 24 hours which were restricted to the lungs (Figure 1C). Neither bacteremia nor bacterial spread into the liver were present at any time investigated confirming a non-bacteremic *S. aureus* pneumonia.

### mCLCA3 modulated a selective pulmonary inflammatory cytokine release in acute staphylococcal pneumonia

**Figure 2:**
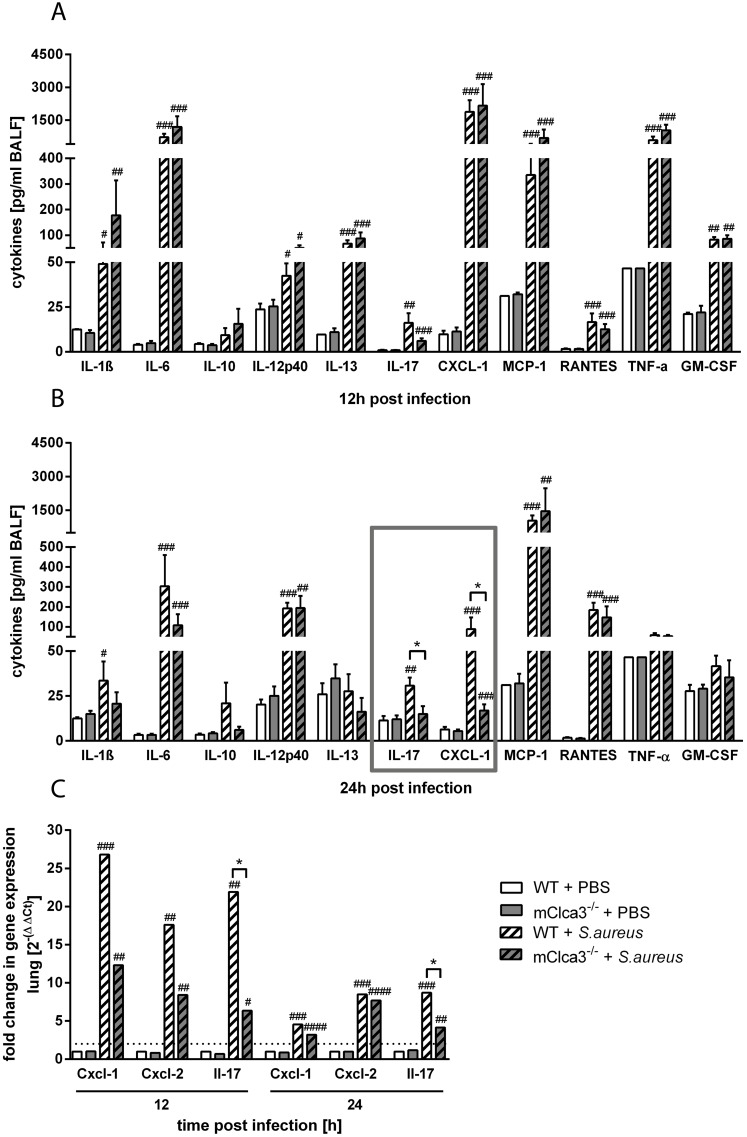
Chemokine CXCL-1 and cytokine IL-17 were significantly decreased in BALF of infected mCLCA3-deficient mice. (A, B) Protein levels of cytokines were measured at 12 hours and 24 hours post infection by multiplex assay technique (IL-1β, IL-6, IL-10, IL-12p40, IL-13, CXCL-1, MCP-1, RANTES, TNF-α, GM-CSF) or by ELISA (IL-17) in BALF. (C) Expression levels of Cxcl-1, Cxcl-2 and Il-17 were quantified by RTq-PCR in the lungs. Dotted line indicates a fold change of 2 as limit for valid statement of elevated parameters. Values are given as mean ± SEM (n = 6 to 8 each group). ^#^p<0.05, ^##^p<0.01, ^###^p<0.001, ^####^p<0.0001 versus PBS control group. *p<0.05 as indicated.

Inflammatory cytokines were quantified on protein level in BALF (IL-1β, IL-6, IL-10, IL-12p40, IL-13, IL-17, CXCL-1, MCP-1, RANTES, TNF-α, GM-CSF) or on mRNA level in lung homogenates (Cxcl-1, Cxcl-2, Il-17) of *S. aureus* infected mice and PBS controls. In infected animals, all cytokine protein levels analyzed, except for IL-10, were increased 12 hours after infection as compared to uninfected mice, independently of genotype (Figure 2A). In contrast, after 24 hours, infected mCLCA3-deficient mice showed a significant decrease of the cytokines CXCL-1 and IL-17 compared to infected wild-type mice. Furthermore, levels of IL-1β, IL-6, IL-13, CXCL-1, TNF-α and GM-CSF slightly declined, whereas levels of IL-12p40, MCP-1 and RANTES increased over time in infected mice (Figure 2B). RTq-PCR analysis of lung homogenates after 12 hours showed a 2.17 fold decrease of Cxcl-1-mRNA and a significant 3.4 fold decrease of Il-17-mRNA in infected mCLCA3-deficient mice compared to infected wild-type mice. Additionally, Cxcl-2-mRNA was decreased by a factor of 2.04 in infected mCLCA3-deficient mice compared to infected wild-type mice. After 24 hours, Il-17 mRNA levels remained significantly decreased in infected mCLCA3-deficient mice compared to infected wild-type mice whereas the mRNA expression of Cxcl-1 and Cxcl-2 of the infected groups showed similar levels. Moreover, at indicated time points, mRNA levels of all parameters investigated were significantly elevated compared to PBS controls (Figure 2C).

### Leukocyte recruitment into *Staphylococcus aureus*-infected lungs was dependent on mCLCA3

**Figure 3:**
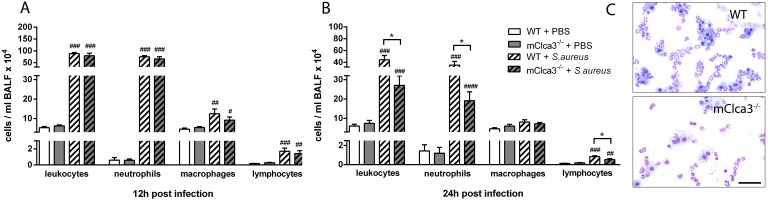
Pulmonary neutrophils and lymphocytes were significantly decreased in mCLCA3-deficient mice 24 hours after infection. (A, B) At 12 hours and 24 hours after infection, leukocytes in bronchoalveolar lavage fluid (BALF) were manually counted and subpopulations were analyzed by fluorescence-activated cell sorter (FACS). Values are given as mean ± SEM (n = 8 each group). ^#^p<0.05, ^##^p<0.01, ^###^p<0.001, ^####^p<0.0001 versus PBS controls. *p<0.05 as indicated. (C) Cytospins from BALF 24 hours post infection were obtained by centrifugation and BALF cell suspension was subsequently stained with May-Grünwald Giemsa. Leukocyte numbers were reduced in BALF from mClca3^−/−^ mice (n = 8 each group). *Bar,* 50 µm.

**Figure 4:**
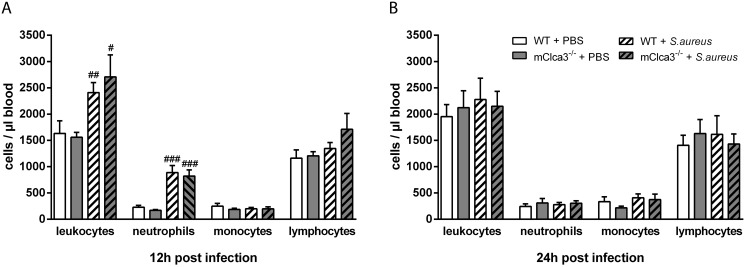
Early infection with *S. aureus* led to a comparable increase in systemic leukocyte response. (A, B) At indicated time points after infection, blood leukocyte numbers and subpopulations were determined by fluorescence-activated cell sorter (FACS). Values are given as mean ± SEM (n = 8 each group). ^#^p<0.05, ^##^p<0.01, ^###^p<0.001 versus the PBS-treated control group.

At indicated time points, leukocyte numbers and subsets were examined in BALF by fluorescent-activated cell sorter (FACS) analysis. Total leukocyte numbers increased significantly within 12 hours in infected animals independently of genotype compared to PBS controls (Figure 3A). Predominantly neutrophils, as well as macrophages and lymphocytes to a lesser extent, contributed to the observed leukocyte influx into the bronchoalveolar space without any genotype difference in numbers. In contrast, 24 hours after *S. aureus* infection, mCLCA3-deficient mice showed significantly decreased numbers of total leukocytes, neutrophils and lymphocytes compared to infected wild-type mice while numbers of macrophages remained equal. In uninfected mCLCA3-deficient mice, leukocyte numbers were comparable to those of uninfected wild-type mice (Figure 3B). Cytospin analysis from BALF cell suspensions of infected mice confirmed the results (Figure 3C). However, only 12 hours after *S. aureus* infection systemic leukocyte response in the blood was altered. Numbers of leukocytes and specifically neutrophils were significantly increased in infected mice compared to PBS controls, however, independently of genotype (Figure 4).

### Infected mCLCA3-deficient mice had reduced protein amounts in BALF without genotype specific changes in pulmonary vascular permeability

**Figure 5:**
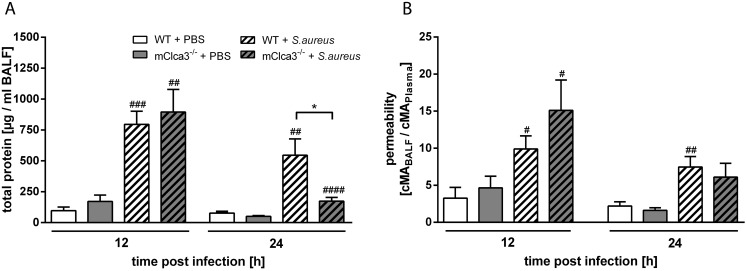
mCLCA3-deficiency led to reduced protein quantities in BALF during infection without altered pulmonary vascular permeability. (A) Total protein of BALF was examined at indicated time points by BCA-assay. (B) Mouse albumin (MA) of transnasally infected or uninfected mice was measured by ELISA in BALF and plasma. Pulmonary vascular permeability was calculated from the MA BALF/plasma ratio. Values are given as mean ± SEM (n = 8 each group). ^#^p<0.05, ^##^p<0.01 versus the PBS control group. *p<0.05 as indicated.

Total protein amount in BALF was determined with the BCA-method. Infected mice had increased quantities of total protein 12 hours after infection with *S. aureus* compared to PBS controls without genotype differences. In contrast, after 24 hours, infected mCLCA3-deficient mice showed a significant 3-fold reduction of BALF proteins compared to infected wild-type mice while PBS controls did not show genotype-specific differences (Figure 5A). As an indicator for the loss of vascular integrity, mouse albumin (MA) concentrations were quantified in BALF and plasma. Pulmonary vascular permeability, assessed by calculating the MA BALF/plasma ratio, was significantly increased in *S. aureus* infected mice compared to PBS controls independently of genotype (Figure 5B).

### *Staphylococcus aureus* infection did not alter mCLCA3 protein and mRNA expression or induce differential regulation of other CLCA family members in mCLCA3-deficient mice

**Figure 6:**
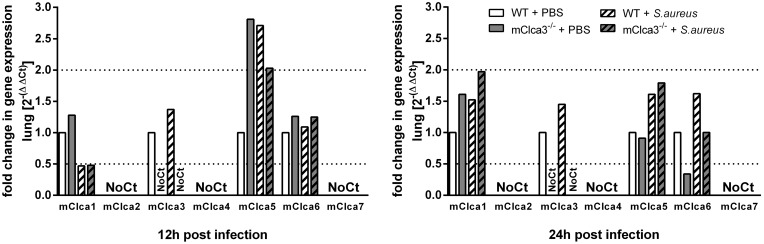
mCLCA3-deficiency in *S. aureus* infection was not compensated by regulation of other CLCA members. Lung expression mRNA levels of mClca1 to 7 were quantified by RTq-PCR. Dotted lines indicate a fold change of 0.5 and 2, respectively, as limits for valid statement of lowered and elevated parameters. Values are given as mean ± SEM (n = 8 each group). Ct, cycle treshold.

Immunoblotting of BALF using mCLCA3-specific antibodies as well as RTq-PCR analysis of mClca3-mRNA in lungs (Figure 6) failed to reveal differences on protein or mRNA levels in infected wild-type mice compared to PBS controls at all time points investigated. Furthermore, no mCLCA3 mRNA (Figure 6) or protein was detected in mCLCA3-deficient mice confirming the knockout status. Additionally, mRNA levels of mClca1 to 7 were measured in lungs of *S. aureus* infected mice and PBS controls by RTq-PCR. mClca1, mClca3, mClca5, and mClca6 were expressed in the lungs, whereas mClca2, mClca4 and mClca7 were not detected. After 12 hours, exclusively mClca5 was increased during infection independently of genotype, therefore no significant changes in expression levels of putative compensatory Clca homologs were observed in the lung (Figure 6).

### Mucus cell number and mucin expression were independent of mCLCA3 during *Staphylococcus aureus* infection

**Figure 7:**
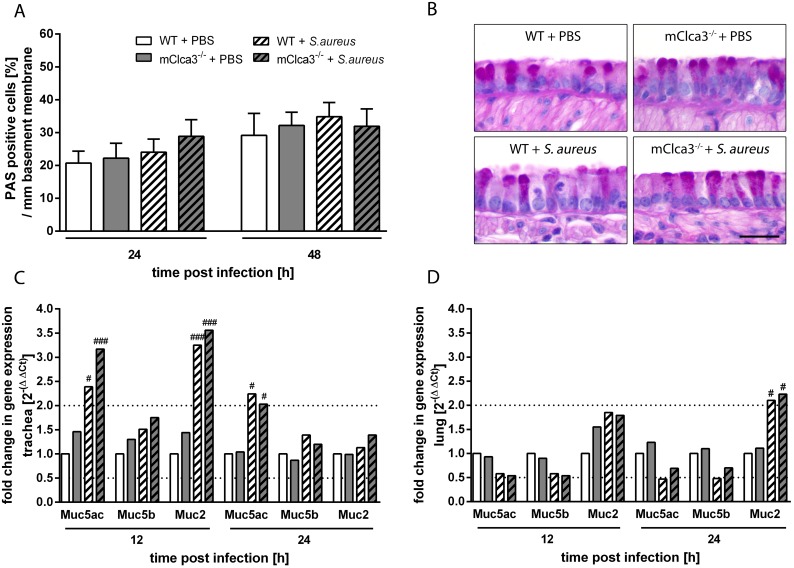
mCLCA3-deficiency had no influence on mucus cell number and mucin expression in infected mice. (A) Mucus cells were quantified by calculating the percentage of periodic acid Schiff (PAS)-positive cells per mm basement membrane. (B) No differences in number or distribution of mucus cells were observed in *S. aureus* infected or uninfected mice. (C, D) Expression levels of Muc5ac, Muc5b and Muc2 were quantified at indicated time points in trachea and lung by RTq-PCR. Dotted lines indicate fold change of 0.5 and 2, respectively, as limit for valid statement of lowered and elevated parameters. Values are given as mean ± SEM (n = 4 each group (A, B), n = 8 each group (C, D)). ^#^p<0.05, ^##^p<0.01, ^###^p<0.001, versus the PBS control group. *Bar* (B), 20 µm.

Quantification of PAS-positive mucus cells per mm basement membrane revealed no differences between infected or uninfected mice independently of genotype at investigated time points (Figure 7A, 7B). Tracheal mRNA levels of Muc5ac and Muc2 were significantly increased 12 hours after infection in mCLCA3-deficient mice and wild-type mice compared to PBS controls, whereas 24 hours after infection only Muc5ac was slightly elevated in the trachea of infected animals (Figure 7C). In contrast, only Muc2 was significantly up-regulated on mRNA level in the lungs of *S. aureus* infected mice, whereas gene expression levels of the other mucin genes tested were not differentially regulated in lung tissue (Figure 7D).

### mCLCA3-deficiency had no effect on histological and morphometric parameters in acute staphylococcal pneumonia

**Figure 8:**
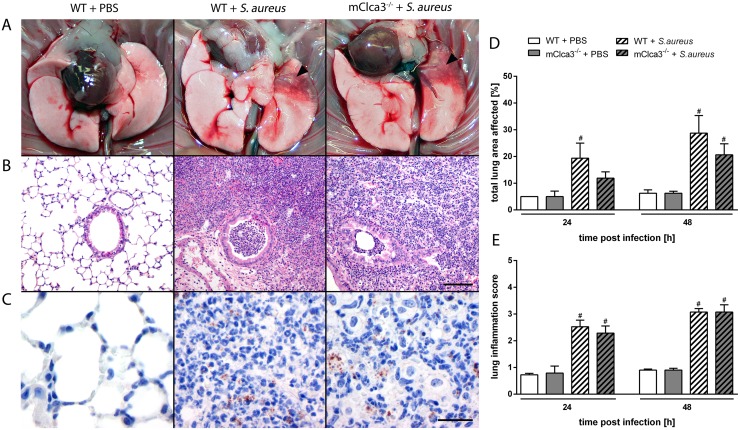
No genotype differences in lung inflammation, lesion distribution or bacterial loads were observed during infection. Mice were transnasally infected with 5×10^7^ *Staphylococcus aureus* Newman and killed at indicated time points. (A) Macroscopic examination revealed deeply red consolidated areas in the infected lungs (arrowhead) in contrast to PBS controls which behaved virtually identical. (B) Subsequently, lungs were fixed, embedded in paraffin and stained with hematoxylin and eosin for histopathological analyses. (C) Evidence of bacteria was assessed by immunohistochemistry with anti-*Staphylococcus aureus* antibody. Uninfected animals (left panel) served as negative controls. *Brown*, 3,3′-diaminobenzidine; *blue*, hematoxylin counterstain. (D) The total lung area affected by inflammation and (E) a lung inflammation score were determined. Values are given as mean ± SEM (n = 4 each group). ^#^p<0.05 versus the PBS control group. *Bar* (B), 100 µm. *Bar* (C), 20 µm.

**Figure 9:**
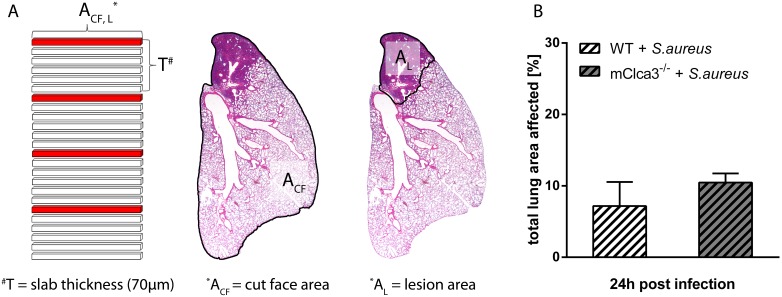
mCLCA3 had no impact on severity or expansion of lung inflammation. (A) 24 hours after infection, left lungs of *Staphylococcus aureus* infected mice were used for quantification of lung lesions by Cavalieri principle. (B) Percentages of estimated lung lesion volumes and total lung volumes of the left lungs were calculated. Values are given as mean ± SEM (n = 4 each group).

Course of pneumonia, lung damage and lesion distribution were further analyzed by pathological investigation. Macroscopical examination revealed first lesions in infected mice independently of genotype as early as 6 hours after infection with progressive exacerbation over time. The upper third of the left lung was predominantly affected. The altered lung tissue was colored deeply red and, in few cases, visible accumulations of suppurative exsudate were present within the pneumonic areas (Figure 8A). Histologically, infected lungs from both genotypes showed a moderate to severe, acute, multifocal, necro-suppurative bronchopneumonia with prominent perivascular edema, multifocal hemorrhage and massive accumulation of neutrophils and macrophages in the consolidated areas (Figure 8B). *S. aureus* was detected by immunohistochemistry using a specific anti-*S. aureus* antibody exclusively in infected animals mostly within macrophages and neutrophils at each investigated time point (Figure 8C). For semiquantification of histologic lesions, total affected lung areas were determined (Figure 8D). Additionally, for evaluation of severity, several parameters were defined (Table 1) and a lung inflammation score was assessed (Figure 8E). No differences between genotypes, neither in severity nor in expansion of lung lesions were observed at indicated time points in *S. aureus* infected mice. Only mild infiltration by macrophages close to the hilum of the lungs was observed in uninfected controls independently of genotype, likely due to the application of PBS. To examine the lung as a 3-dimensional structure and to warrant complete measurement of lung lesions, volume was estimated by Cavalieri principle in *S. aureus* infected lungs. Whole left lungs were cut into consecutive sections of equal thickness and each seventh slide was analyzed at indicated time points (Figure 9A). No genotype differences, neither in volume of examined lungs nor in percentages of lesions were observed (Figure 9B).

## Discussion

Several previous studies have analyzed the role of hCLCA1 and its murine ortholog mCLCA3 which were overexpressed in complex inflammatory airway diseases, primarily focusing on mucus cell regulation and induction of mucus cell metaplasia [9], [13], [14], [17], [37]–[39]. More recently, it has been reported that both proteins may have an impact on airway inflammation and regulation of innate immune responses [13]–[16].

In the present study, we describe an inflammatory phenotype of mCLCA3-deficient mice in an acute bacterial lung infection model with *Staphylococcus aureus* Newman. mCLCA3-deficiency was associated with decreased protein-levels of CXCL-1 and IL-17 in the BALF compared to wild-type mice 24 hours post infection. CXCL-1 is a murine homolog to human CXCL-8 and a potent chemoattractant for neutrophils [40], [41] and IL-17 mediates proinflammatory responses primarily by inducing the expression of other cytokines and chemokines, including CXCL-8 [42]. In contrast, all other cytokines tested (IL-1β, IL-6, IL-10, IL-12p40, IL-13, MCP-1, RANTES, TNF-α and GM-CSF) were significantly increased after infection, independently of mCLCA3-deficiency, pointing towards selective modulation of a specific subset of cytokines in mCLCA3-deficient mice after bacterial challenge. Consistent with our protein data, markedly decreased mRNA-levels of Cxcl-1 and Il-17 were observed as early as 12 hours post infection. Similarly to Cxcl-1, Cxcl-2, another murine CXCL-8 homolog [43], showed a similar transcriptional regulation in challenged mCLCA3-deficient mice. The reduced induction of these cytokines on the mRNA and protein levels points towards aberrant transcriptional regulation in mCLCA3-deficient mice after *S. aureus* challenge. As a consequence of the low CXCL-1 levels, neutrophilic recruitment to the site of infection was decreased after 24 hours. However, as an alternative explanation for reduced leukocyte numbers in bronchoalveolar spaces, capillary-alveolar transmigration may also have been reduced. Although we failed to observe differences in plasma protein extravasation, this additional effect cannot be fully excluded. Besides neutrophils, lymphocytes were also significantly reduced in the BALF of mCLCA3 deficient mice 24 hours after infection. The cytokines CXCL-1 and IL-17 found dysregulated in this study are neither known to be chemoattractants for lymphocytes nor inducers of lymphocyte proliferation. It is more likely that a yet unidentified factor influencing lymphocyte recruitment and/or proliferation may also be modulated after *S. aureus* challenge in the context of mCLCA3 deficiency.

We hypothesized that attenuation of coordinated leukocyte recruitment in mCLCA3-deficient mice was accompanied by reduced lung barrier destruction. However, the albumin BALF/plasma ratio, quantified for determination of pulmonary endo-epithelial barrier failure, was not reduced in mCLCA3-deficient mice. It thus appears likely that reduction of proteins from viable and degenerate leukocytes due to decreased bronchoalveolar leukocyte influx was the main contributor to the diminished total protein content in BALF of mCLCA3-deficient mice.

The predominant cell types in the lung expressing the two CXCL-8 homologs CXCL-1 and CXCL-2 during bacterial infections are alveolar macrophages and pulmonary epithelial cells [41], [44]. Although subsets of T-lymphocytes are the primary source of IL-17, it was previously demonstrated that alveolar macrophages are also competent of secreting IL-17 [45], [46]. The altered transcriptional regulation of the CXCL-8-homologs and IL-17 identified in this study is in line with the recently hypothesized additional role of CLCA-proteins as signaling molecules in inflammation [10], [15]. Specifically, these studies demonstrated that hCLCA1, the human ortholog of mCLCA3 with virtually identical structure and cellular expression pattern, acts as a signaling molecule that induces mucus gene transcription via a downstream MAPK-13 signaling pathway [10]. Furthermore, hCLCA1 activates alveolar macrophages resulting in pro-inflammatory cytokine induction, including CXCL-8 [15]. It thus appears reasonable to assume that mCLCA3 may have a similar effect on mouse alveolar macrophages after bacterial challenge with *S. aureus* via murine CXCL-8 homologs and IL-17. However, we cannot exclude that the reduced murine CXCL-8 homolog expression in mCLCA3-deficient mice is a downstream event due to the aberrant induction of IL-17, which is a known inducer of CXCL-8 [42], [47] instead of being directly regulated by mCLCA3.

A link between IL-17 and mCLCA3 has recently been suggested. In that study, neutralization of IL-17 in a murine viral lung infection model decreased mRNA-levels of mClca3 and also decreased Cxcl-1-mRNA levels with reduced infiltration of neutrophils into the bronchoalveolar space [48]. Further studies should elucidate the relation between IL-17 and mCLCA3, especially the effect of mCLCA3 on IL-17-expressing cell types.

In contrast to our study, a previous study revealed that mCLCA3-deficient mice challenged with ovalbumin or LPS had increased numbers of neutrophils and that LPS-treated mice additionally showed enhanced CXCL-1 protein levels without alterations in CXCL-2 and IL-17 protein levels [13]. This seemingly contradictory result may be due to the different inducers of inflammation (LPS vs. bacterial infection) and/or different mouse strains used (129SvEvBrd vs. C57BL/6J). Moreover, LPS is an endotoxin from Gram-negative bacteria whereas in this study we used a Gram-positive bacterial species which could also account for the seemingly contradictory results.

The acute *S. aureus* pneumonia in mice is an appropriate model for studying innate immune responses [18], [49]–[51]. However, based on the well established role of CLCA-proteins in Th2 cytokine driven inflammatory airway diseases with increased mucus production and mucus cell metaplasia [9], [13], [14], [17], [37]–[39], we also analyzed the effect of mCLCA3 on mucus cells and mucin gene expression in this acute *S. aureus* pneumonia. No differences in the extent of mucus cell metaplasia or mucin gene induction were observed after infection with *S. aureus* between mCLCA3-deficient and wild-type mice. The increase of Muc5ac mRNA levels without significant changes in Muc5b gene expression in infected mice compared to PBS controls are consistent with previous reports [9], [10], [17]. In addition, Muc2 mRNA levels were found to be increased in *Staphylococcus aureus* infected animals compared to PBS controls independent of genotype corresponding to previously reported results of lipoteichoic acid (LTA) from gram positive bacteria strongly inducing Muc2 expression [52]. The induction of mucus production under bacterial challenges is thus a known innate immune response irrespective of the mCLCA3 status.

Interestingly, mCLCA3 independent mucin regulation in airway diseases shows a discrepancy between mice and humans [10]. It was previously hypothesized that upregulation of other members of the murine CLCA family may possibly compensate for the lack of mCLCA3 [9], [10], [17]. In addition to mCLCA3, the murine CLCA5, -6, and -7 are potential candidates to drive mucus cell metaplasia in mice [11]. Therefore, we analyzed the lungs regarding a possible compensatory differential regulation of other murine CLCA homologs. mClca1, mClca3, mClca5, and mClca6 were expressed, whereas mClca2, mClca4 and mClca7 were not detected. Of these, only mClca5 was increased during infection, however, independently of genotype. Thus, no differentially regulated and putatively compensatory CLCA member with regard to mucus cell regulation was found, even under challenged conditions which is in line with previous reports [17], [53].

In contrast to the observed reduction in neutrophil infiltration, our pathologic examination failed to reveal differences in lung inflammation or lung lesion expansion between genotypes despite using up-to-date morphometric methods [34]. Infected animals developed an acute, marked, suppurative and necrotizing bronchopneumonia with consolidation and destruction of inflamed areas. Due to this tissue destruction, an exact separation between lung parenchyma and alveolar spaces was impossible and histological quantification of cell types in the two separate compartments that could have confirmed the results of BALF analyses was impossible. Furthermore, no differences in clinical course (physical constitution, behavior, body temperature, changes in body weight) or lung bacterial loads were observed following infection, indicating that the effect of mCLCA3 on the molecular and subsequent cellular responses had no obvious impact on clinical and pathological outcome at the times investigated. Obviously, additional determinants other than leukocyte number decide on the overall severity of pneumonia and clinical outcome.

In conclusion, our data suggest that mCLCA3 modulates the cellular leukocyte recruitment via IL-17 and CXCL-1 in acute *S. aureus* pneumonia. Lack of mCLCA3 led to a selectively decreased induction of IL-17 and CXCL-8 homologs and decreased numbers of neutrophils and total protein in BALF in *S. aureus* infected mice. During bacterial infection, no differences were observed in mucin regulation and no other mCLCA family members were differentially regulated. Thus, mCLCA3 seems to have an impact on the early innate immune response via direct or indirect induction of select cytokines during *S. aureus* infection.

Further studies should characterize the specific cytokine pathways and the main target cells activated by mCLCA3 under physiological condition and infection. Prospectively, mCLCA3 may even become a potential therapeutic target for the modulation of inflammation in lung infections.

## Supporting Information

Table S1 Quantitative Real Time RT-PCR: Sequences and Specifications.
(DOCX)
