# Supplementary material for: mCLCA3 Modulates IL-17 and CXCL-1 Induction and Leukocyte Recruitment in Murine Staphylococcus aureus Pneumonia
Source: PLoS One. 2014 Jul 17;9(7):e102606. doi: 10.1371/journal.pone.0102606 (PMC4102496; doi:10.1371/journal.pone.0102606)
Supplement: Table S1 — Quantitative Real Time RT-PCR: Sequences and Specifications. (DOCX) [file pone.0102606.s001.docx]

SUPPORTING INFORMATION

Table S1

**Quantitative Real Time RT-PCR: Sequences and Specifications**

| Gene | Genbank  Accession no. | Oligonucleotide Sequences (5’-3’) | Amplicon  Size, bp | Annealing Temp., °C |
| --- | --- | --- | --- | --- |
| *mClca1* | NM_009899 | Primer (upstream) GTGGACCAGCCTTTCTACATGTCTAG  Primer (downstream)TGTGACACAGTTGCCTCTCTCA  TaqMan Probe FAM-ATCACTGGCACCAATGTGGTTCACAAC-BHQ | 114 | 60 |
| *mClca2* | NM_030601 | Primer (upstream) GGACCGGCCTTTCTACATTTCTAG  Primer (downstream)CACAGGCAAGAAGGTGGTCCACGA  TaqMan Probe FAM-CACACAGCTGCCTCTCTGACA-BHQ | 109 | 60 |
| *mClca3* | NM_017474 | Primer (upstream) GAAATTCCCCAGCCCTGTAACAG  Primer (downstream)CAAGCATTCGCCAAGGAGCCTCGCC  TaqMan Probe FAM-TGCGCACCTGCTCCGTTATC-BHQ | 144 | 60 |
| *mClca4* | NM_139148 | Primer (upstream) ACTAACCTAATAAGGATCATCAATGA  Primer (downstream)CTCCTACCTAGCGATCAGCACAAAGC  TaqMan Probe FAM-AGTTCCGCCATTTGGGTATT-BHQ | 81 | 60 |
| *mClca5* | NM_178697 | Primer (upstream) ACGATGACCGGAAGCTGCTG  Primer (downstream)CCTGCCGACCGCCGTGTCCAC  TaqMan Probe FAM-ACCACCTCAAAGCCTTTCTTAACC-BHQ | 100 | 60 |
| *mClca6* | NM_207208 | Primer (upstream) CATAATAAAAAGTGCAATTACAG  Primer (downstream)CTTGGGAAGTAATCAGCACTTCTG  TaqMan Probe FAM-AGGGTGACGTCTCCAT-BHQ | 91 | 60 |
| *mClca7* | NM_001033199.3 | Primer (upstream) ATGGAGACACCACCGG  Primer (downstream)CTTCTTCTCCCTGCTGAGGATCAG  TaqMan Probe FAM-TCTATTCAGACGGTCAGAAGAAC-BHQ | 114 | 60 |
| *Muc5ac* | NM_010844.1 | Primer (upstream) GCGTGGAGAATGAAAAGTATGCT  Primer (downstream)CCCAGTGCCACGCCACCGTG  TaqMan Probe FAM-CATACATGCAGTTCGAGAAGAAG-BHQ | 120 | 60 |
| *Muc5b* | NM_028801 | Primer (upstream) GCACGTAAATGCGACTGTCT  Primer (downstream)TATCCAAGTACTCCATGGAGGCCC  TaqMan Probe FAM-ATGGACCTTGCTCTCCTGAC-BHQ | 154 | 60 |
| *Muc2* | NM_023566 | Primer (upstream) GAGGCAGTACAAGAACCGGA  Primer (downstream) TTCGGCTCGGTGTTCAGAG  TaqMan Probe FAM-CCATTGAGTTTGGGAACATGC-BHQ | 104 | 60 |
| *Cxcl1* | NM_008176.3 | Primer (upstream) GATGCTAAAAGGTGTCCCCA  Primer (downstream)AGACTGCTCTGATGGCACCT  TaqMan Probe FAM-GTCAGAAGCCAGCGTTCAC-BHQ | 83 | 60 |
| *Cxcl2* | NM_009140.2 | Primer (upstream) CTGAACAAAGGCAAGGCTAACT  Primer (downstream)ACCTGGAAAGGAGGAGCCT  TaqMan Probe FAM-CTTTGGTTCTTCCGTTGAGG-BHQ | 73 | 60 |
| *Il-17A* | NM_010552 | Primer (upstream) GCTCCAGAAGGCCCTCAGA  Primer (downstream)CTCTCCACCGCAATGAAGACCCTGA  TaqMan Probe FAM-AGCTTTCCCTCCGCATTGA-BHQ | 142 | 60 |
| *Ef1a* | NM_010106.2 | Primer (upstream) AAAAACGACCCACCAATGG  Primer (downstream)GGCCTGGATGGTTCAGGATA  TaqMan Probe FAM-AGCAGCTGGCTTCACTGCTCAGGTG-BHQ | 67 | 60 |
| *B2m* | NM_009735.3 | Primer (upstream) ATTCACCCCCACTGAGACTGA  Primer (downstream)CTCGATCCCAGTAGACGGTC  TaqMan Probe FAM-TGCAGAGTTAAGCATGCCAGTATGGCCG-BHQ | 86 | 60 |
| *Gapdh* | NM_008084.2 | Primer (upstream) TCACCACCATGGAGAAGG  Primer (downstream)GCTAAGCAGTTGGTGGTGCA  TaqMan Probe FAM-ATGCCCCCATGTTTGTGATGGGTGT-BHQ | 169 | 60 |

E
